# Practical Notes on Diagnosis and Treatment

**Published:** 1906-07-21

**Authors:** 


### Practical Notes on Diagnosis and Treatment.

**Pediculosis.**

An excellent and cleanly plan of treating pediculosis of the body is to make the patient wear a piece of sulphur in a small muslin bag slung from the neck.—*Dr. H. G. Adamson.*

**The Memory in General Paralysis.**

Sometimes in advanced stages of general paralysis there is a quite remarkable retention of memory, whilst in other forms of progressive mental decay, associated for example with alcoholism or senility, loss of memory may be the chief characteristic.—*Dr. Savage.*

**Pigmentation in Skin Diseases.**

Of course, a large proportion of syphilides do produce pigmentation, but syphilis is by no means alone in that respect, and pigmentation should never lead you to the diagnosis of an eruption as specific. Especially is this the case with lichen planus, where, as a rule, the pigmentation is deeper than in an average syphilitic eruption.—*Dr. Radcliffe Crocker.*

